# Humeral Shaft Fractures Secondary to Hand Grenade Throwing

**DOI:** 10.1155/2013/962609

**Published:** 2013-04-21

**Authors:** Bahattin Kerem Aydin, Ramazan Akmese, Mustafa Agar

**Affiliations:** ^1^Selcuk University, Faculty of Medicine Orthopaedics and Traumatology Clinic, 42070 Konya, Turkey; ^2^Ankara Ataturk Educational Hospital Orthopaedics and Traumatology Clinic, 06100 Ankara, Turkey; ^3^Denizli State Hospital Orthopaedics and Traumatology Clinic, 20110 Denizli, Turkey

## Abstract

A series of five cases were presented in which similar fractures of the shaft of the humerus occurred during the hand grenade throwing activity during the military education. All the fractures were in the 1/3 distal humeral shaft, and butterfly fragments were accompanying in two soldiers. All the fractures healed without any clinical complications with conservative treatment. The mechanism of the fracture is discussed with reference to the recent literature.

## 1. Introduction

Humerus fractures are generally secondary to the direct trauma [1, 2]. Fractures of the shaft of the humerus as a result of muscular violence are uncommon. Spiral fractures of the humerus have been reported in throwing sports such as baseball, softballs, handballs, javelins, and hand grenades [3–5]. This type of fractures is also reported among the hand wrestlers [6]. Sometimes, especially in teenagers and geriatric population, this type of violence can cause spiral fractures who has oncologic bone disease. Throwing fractures of the humeral shaft are controversial whether they are related to a stress fracture or a sudden intense torsional load. Stress fracture patients generally have complaints of arm pain and repeating throwing activity before the fracture. But in torsional stress group, there is always a history of sudden intense torsional activity just before the fracture.

 In the present study, spiral humeral shaft fractures are secondary to the hand grenade throwing in five military recruits. The causes of these fractures and the literature related to the hand grenade throwing were also reviewed.

## 2. Materials and Methods

Between August 2008 and January 2009, 5 male military recruits were admitted to the Emergency Department of Ağrı Military Hospital with the right humerus shaft fractures during hand grenade throwing training period. Average patient age was 20.2 years (range 19–22). All the patients were right-handed, and none of them had an experience in throwing sports before their military obligation. The recruits reported that they used the maximum strength when throwing the hand grenade. According to their history, all the fractures occurred just before the hand grenade release.

**Figure 1:**
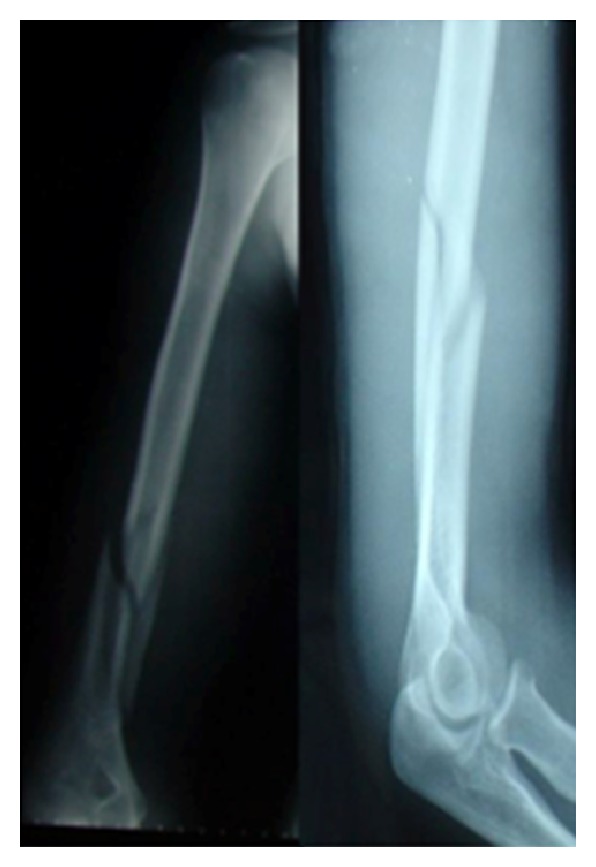
X-rays at the first application to the emergency service.

All fractures were closed and extra-articular. All the fractures were at the junction of the middle and distal third of the humeral shaft (Figure 1). Two of them had a butterfly fragment. No patient had a neurovascular injury.

**Figure 2:**
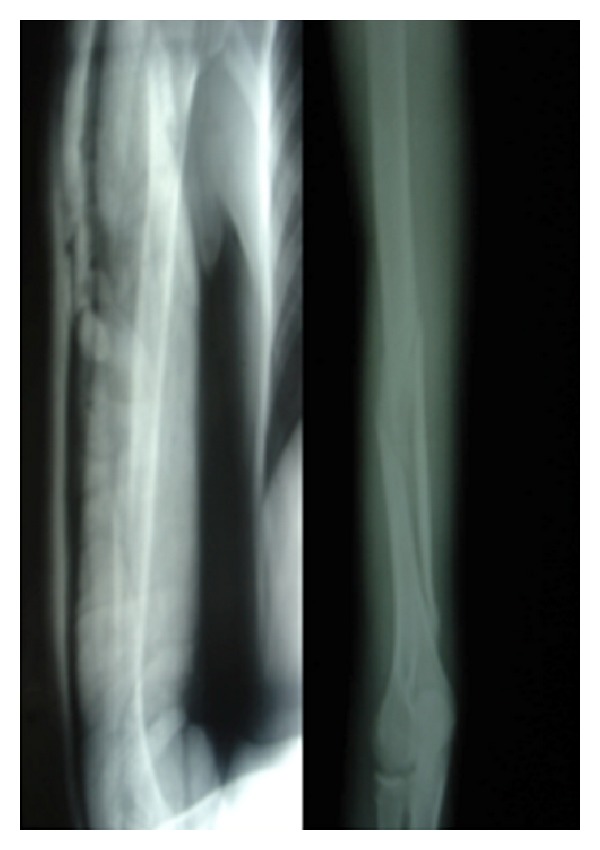
X-rays with U-splint and just after removal.

**Figure 3:**
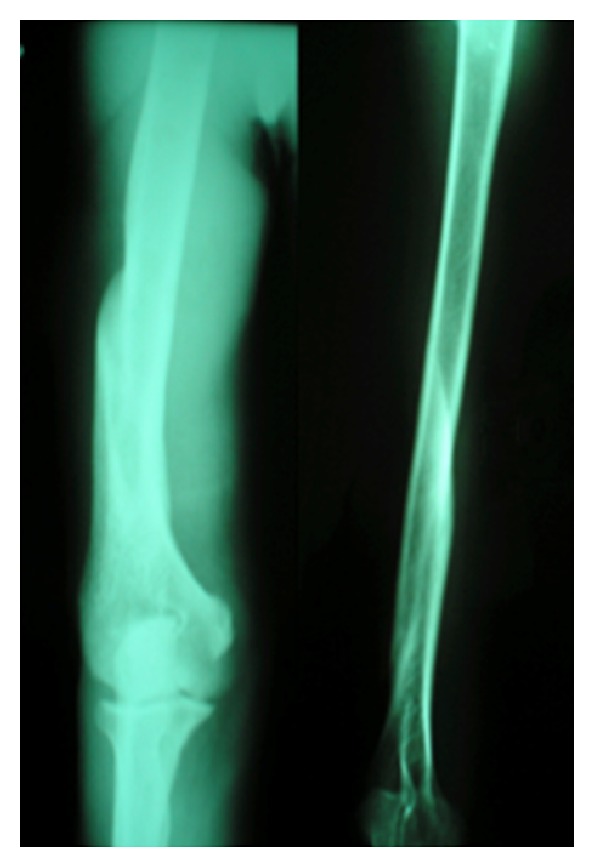
X-rays after brace removal.

All patients were admitted to the clinic on the day of injury. Initial fracture stabilization was achieved with U-splint and the Velpeau bandage for all patients. Patients were systematically examined for accompanying any musculoskeletal disease. Because pathologic fracture was not suspected on plain radiographs, any further imaging techniques were not performed. On radiographs, average varus-valgus angulation was 12′ [7–15], and anterior-posterior angulation was 11.2′ [9–13]. All the patients underwent nonsurgical treatment. Three-week U-splint and Velpeau bandage and then custom-made prefabricated functional brace were applied (Figure 2). Average time to union time was 10.6 [9–13] weeks. Functional examination according to the Hunter Classification was G5 [7]. No patient had lack of elbow motion. No patient required formal physical therapy following brace removal (Figure 3). No patient had radial nerve palsy during treatment or due to entrapment in the callus of healed fracture.

## 3. Discussion

There are several cases of humerus fracture during the throwing activity. Several theories exist for the cause of this type of fracture, including uncoordinated muscular antagonism, lack of a regular exercise program, inadequate throwing technique, and muscle fatigue [8, 9]. And, also, stress fracture can be a problem with the history of rhythmic, repeated, and subthreshold exercise as overuse injuries. However, it is difficult to determine the independent effect of each of these factors.

 Allen reported one humeral shaft fracture that resulted from pitching. He described this as the “window of vulnerability” during the early stages of bone remodeling [10].

 Branch et al. reported 12 humeral shaft fractures in 12 baseball players, and they thought that the lack of exercise period and prolonged layoff periods are the main causes for this type of fractures [8].

 Ogawa and Yoshida suggested that this type of humerus fractures is secondary to the practice limitations, and they presented this as an external rotation fracture in 90 baseball players [9].

 Throwing humeral shaft fractures can be secondary to the stress loading as called stress fracture. At this situation, there must be pain at the rest, and, also, there must be a history of repeating exercise program. Throwers who had fracture with no previous experience of throwing generally had no prodromal pain as in this study.

 The fracture site and type support the suggestions of Ogawa and Yoshida that these fractures occur due to mainly external rotation force on the distal humerus at the acceleration throw phase, as the proximal end internally rotates [9].

 In military education of hand grenade throwing, recruits are told to extend the elbow during the entire acceleration phase. This style does not create an external rotation force on the distal humerus, and only the proximal end internally rotates. In a faulty throwing style, if the elbow is flexed at the early acceleration throw phase, the distal humerus is exposed to external rotation force as the elbow is extended at the late acceleration phase. This antagonism of rotational torques, because of faulty throwing style, is the main cause of throwing fractures in military recruits [11].

 Malignant metastatic tumours are the most neoplasms of bone. These lesions whether metastatic or primary or malignant or benign can be the reason for humeral pathological fractures. As humerus is the second most frequently involving metatstatic lesions, patients ages generally are over 50 years old [12]. Benign lesions are generally seen in the teenager population at the proximal third of the humerus. Most authors agree that as long as the plain radiographs do not show any evidence of pathological bone, further workup is not indicated [13].

 In extra-articular humerus shaft fracture treatment modalities, the first choice is nonsurgical treatment methods; we used to begin the treatment with U-splint and the Velpeau bandage for 3 weeks, and after then, we applied functional brace as the Sarminento brace. This treatment method has low morbidity and high success rate.

 Rotational deformities decrease at the fracture site by contraction of the flexors and extensors [14]. Angulatory deformities of the humeral shaft up to 25′ can be tolerated functionally and cosmetically because of the large soft tissue mass around the humerus and range of movement of the adjacent joints [15].

## 4. Conclusion

 Whatever the condition of the humeral shaft before an overt fracture, the true cause of throwing fracture of the humerus is a rotational force at the acceleration phase of the throw. Almost all the throwing fractures, as in this present study, are spiral fractures, with or without butterfly fragment, and the course of the fracture line shows that they are external rotation fractures. The fracture site usually is at the junction of the middle and distal third of the humerus [8–10]. Conservative methods are the first choice of treatment as it offers good functional and cosmetically results and also low cost with high union rate.
